# Protein Subcellular Relocalization Increases the Retention of Eukaryotic Duplicate Genes

**DOI:** 10.1093/gbe/evt183

**Published:** 2013-11-20

**Authors:** S. Ashley Byun, Sarabdeep Singh

**Affiliations:** ^1^Department of Biology, Fairfield University; ^2^National Center for Biotechnology Information, National Library of Medicine, National Institutes of Health, Bethesda, MD

**Keywords:** duplicate genes, protein subceulluar relocalization, genome evolution, death rates

## Abstract

Gene duplication is widely accepted as a key evolutionary process, leading to new genes and novel protein functions. By providing the raw genetic material necessary for functional expansion, the mechanisms that involve the retention and functional diversification of duplicate genes are one of the central topics in evolutionary and comparative genomics. One proposed source of retention and functional diversification is protein subcellular relocalization (PSR). PSR postulates that changes in the subcellular location of eukaryotic duplicate proteins can positively modify function and therefore be beneficial to the organism. As such, PSR would promote retention of those relocalized duplicates and result in significantly lower death rates compared with death rates of nonrelocalized duplicate pairs. We surveyed both relocalized and nonrelocalized duplicate proteins from the available genomes and proteomes of 59 eukaryotic species and compared their relative death rates over a Ks range between 0 and 1. Using the Cox proportional hazard model, we observed that the death rates of relocalized duplicate pairs were significantly lower than the death rates of the duplicates without relocalization in most eukaryotic species examined in this study. These observations suggest that PSR significantly increases retention of duplicate genes and that it plays an important, but currently underappreciated, role in the evolution of eukaryotic genomes.

## Introduction

For more than 40 years, it has been widely accepted that gene duplication is an important process underlying the evolution of new genes (Ohno 1970). With increasing availability of genomic data over the last 15 years, there has been renewed interest in this aspect of genome evolution, specifically with regards to the various evolutionary mechanisms involved with the retention and functional diversification of duplicate genes or paralogs (Zhang 2003; Innan and Kondrashov 2010). Some of the more well-known mechanisms of retention and diversification include neofunctionalization (Ohno 1970) and subfunctionalization (Hughes 1994; Force et al. 1999), both of which have been reviewed at great length in the literature (Innan and Kondrashov 2010). Recently, protein subcellular relocalization (PSR) was proposed as a means by which duplicate genes can potentially evolve novel functions through changes in the localization of their proteins within the cell (Byun and Geeta 2007; Byun et al. 2009). The basic premise behind PSR is that changes in a protein’s subcellular location can cause it to take on new or modified roles within the cell. Such functional changes due to subcellular relocalization have been observed in biochemical studies (Bizily et al. 2003; Lessering et al. 2004; Heilmann et al. 2004). PSR postulates that if such functional changes happen to a duplicate protein and the change is advantageous, the duplicate gene may be retained and ultimately lead to the evolution of a new gene.

The N-terminal peptide (NTP) is one of the best understood signals responsible for protein subcellular localization (Kaiser et al. 1987; Bannai et al. 2002). The NTP, a short degenerate sequence of approximately 13–85 amino acids located at the N-terminus of a protein, specifies its location within the eukaryotic cell. Once the protein is delivered to its correct subcellular location, the NTP is typically cleaved off and degraded, and therefore does not participate directly in mature protein function (Bannai et al. 2002). Therefore, changes to the NTP sequence can cause proteins to relocate without changing the actual sequence of the mature protein. In some instances, even minor changes to the NTP, such as a single nucleotide subsititution, are potentially capable of altering protein localization (Byun and Geeta 2007).

Over the past few years, several studies have supported the idea that PSR plays a key role in the evolution of duplicate genes in eukaryotes such as *Saccharomyces* (Marques et al. 2008) and humans (Wang et al. 2009). However, to date, no large-scale study has been undertaken to evaluate PSR as a universal mechanism of general eukaryotic duplicate gene evolution. Although a study comparing singleton and duplicate genes in *Saccharomyces* and *Schizosaccharomyces* found no significant difference in the PSR rate (Qian and Zhang 2009), it does not negate the importance of PSR as an evolutionary mechanism for duplicate genes. Rather, it suggests that PSR may play a role in the evolution of orthologous genes as well.

In this study, we examined duplicate gene pairs and their corresponding proteins compiled from the complete genomes and proteomes of 59 metazoan, single-celled eukaryotes, plant, algal, and fungal species to compare the retention (as measured by death rates) of relocalized duplicates and nonrelocalized duplicates over a range of Ks (number of synonymous substitutions per synonymous sites) values 0 to 1. Using the Cox proportional hazard model to compare death rates among over 700,000 relocalized and nonrelocalized duplicate gene pairs, we found compelling evidence to suggest that PSR duplicates have significantly lower death rates than duplicates, which do not relocalize. This observation, which suggests that relocalization significantly increases retention of duplicate genes, is consistent with the idea that PSR plays an important role in the evolution of duplicates and eukaryotic genomes.

### Results and Discussion

A total of 7,16,917 duplicate gene pairs and their corresponding proteins were identified and analyzed from 59 different fungal, metazoan, green plant/green algae, and basal eukaryotic species (supplementary table S1, Supplementary Material online). For each species, we determined the total number of duplicate genes, and then calculated the proportion of each genome that was duplicated (supplementary table S1, Supplementary Material online). To simplify reporting of these data, we placed each of these species in one of the following categories: Fungi, Metazoan, Plants, Algae, and Basal Eukaryotes (single-celled protists) and summarize the data in table 1. The results of our analyses were consistent to what has been documented from other studies. For example, we predicted the percentage of duplicate genes in *Homo sapiens* and *Arabidopsis thaliana* was 32.6% and 50.7%, respectively. Although our estimates appear to be more conservative, they are largely consistent with predicted values of 38% for *H. sapiens* by Li et al. (2001) and 65% for *A. thaliana* by *Arabidopsis* Genome Initiative (2000). Estimates were also consistent with that of Gu et al. (2000) for *Saccharomyces*, *Drosophila*, and *Caenorhabditis elegans*. They estimated the total number of protein families in each species to be 530, 674, and 1,219, respectively; our estimates were 371, 644, and 1,283. The overall consistency of our estimates with those of other studies supports the validity of our method/algorithm of identifying paralogs.

**Table 1:** Average Proportion of the Genome Duplicated in Major Eukaryotic Groups

Group	Average Proportion of Duplication
Fungi/algae	0.13 ± 0.05
Plants	0.37 ± 0.15
Metazoan	0.25 ± 0.11
Basal eukaryote	0.15 ± 0.090

For each duplicate protein pair identified from each eukaryotic species, the subcellular location was predicted using MultiLoc2. We chose MultiLoc2 because of its ability to predict localization in more subcellular compartments and in a greater variety of species groups than other comparable predictors. Furthermore, MultiLoc2 has shown higher accuracy than similar prediction programs through its incorporation of phylogenetic profiles and GO (Blum et al. 2009). It has been documented that some proteins exhibit dual targeting, which can complicate predictions of subcellular localization made by targeting software (Baudisch et al. 2013). We minimized this potential problem by not focusing on predicting specific subcellular locations of duplicate proteins but rather focsuing on whether they were predicted to be in the same or different locations. In this study, we were not interested in predicting the specific subcellular location of duplicate protein pairs. Rather, we focused on whether they were predicted to be the same or different. We categorized duplicate pairs as either relocalized (duplicate proteins with different predicted subcellular locations) or nonrelocalized (duplicates with identical predicted subcellular locations). We used these estimates to calculate the frequency of relocalized duplicate gene pairs (supplementary table S1, Supplementary Material online, %RDG) and summarize the data in table 2. The percentage of relocalized gene pairs ranged from 21.3% in metazoa to 29.1% in basal eukaryotes. The upper range of relocalized duplicates in individual species were found in rice (*Oryza sativa* 67,697/1,91,985 = 35.3%), and platypus (*Ornithorhynchus anatinus* 2,360/4,116 = 57.3%). The lower range was represented by *Drosophila melanogaster* (173/1,679 = 10.3%), horse (*Equus caballus* 1,234/13,753 = 15.7%), and the trypanosomatid *Leishmania major* (130/1,768 = 13.1%). Although the predictive nature of MultiLoc2 is a limitation of this study, we were encouraged to find that its predictions of subcellular localization were consistent with an empirically derived estimate for *S. cervisiae*. Our predicted estimate of 28.6% (562/1,966) for *S. cervisiae* falls within the 24–37% range empirically determined by Marques et al. (2008).

**Table 2:** Frequency of Relocalized Duplicates within Major Eukaryotic Groups

Group	RD	TND	FRD
Fungi	962	3,453	0.28
Plants/algae	164,663	528,618	0.31
Metazoan	37,491	175,899	0.21
Basal eukaryotes	2,607	8,947	0.29

Note.—RD, relocalized duplicates; TND, total number of duplicates; FR, frequency of relocalized duplicates. For each eukaryotic group, the frequency of PSR among duplicates is high. The total number of relocalized duplicate proteins are based on subcellular locations predicted by MultiLoc2.

For each species, we calculated the hazard ratio (death rate for nonrelocalized/death rate for relocalized) for duplicate pairs with Ks values ranging from 0 < Ks < 1 (supplementary table S2, Supplementary Material online, for full list of all ratios) using the Cox proportional hazard model. We chose a cut off of Ks = 1 to minimize potential errors associated with multiple hits (Li 1997) and potential multiple relocalizations at higher Ks values. The aim of using the Cox proportional hazard model was to compare the death rates of nonrelocalized and relocalized duplicate pairs by estimating the hazard ratio associated with them. Hazard ratios more than 1 indicate a higher death rate of nonrelocalized duplicates relative to the death rate of relocalized (PSR) duplicates. The hazard ratios for eight species ranging from *V. carteri* to *H. sapiens* are shown in table 3 as examples of our total data set (supplementary table S2, Supplementary Material online). The hazard ratios can be interpreted as follows: a hazard ratio of 1.58 for *V. carteri* with 0 < Ks < 0.05 (table 3) mean that nonrelocalized duplicate genes have a 58% higher hazard rate or death rate as compared with relocalized duplicates. Within individual species, hazard ratios varied as a function of Ks. In other words, death rates appeared to fluctuate with the duplicate gene’s relative age. Even in relatively young duplicates (Ks ≤ 0.05), 33.9% (20/59) of all species were observed to have hazard ratios significantly greater than 1 compared with 6.8% of species that showed the reverse (hazard ratio < 1). The observation that death rates in relocalized duplicates was significantly lower than nonrelocalized duplicate pairs at a Ks ≤ 0.05 for 34% of the species we examined, suggests that PSR may influence paralog retention during the earlier stages of duplication in some eukaryotic species. This is particularly interesting given that evolutionary forces, which act in the early stages following duplication, may be crucial in determining the ultimate fate of duplicated genes (Moore and Purugganan 2003). Hazard ratios were also not consistent between species (table 3). However, when hazard ratios were examined over a large number of eukaryotes from 0 < Ks < 1, it was apparent that, overall, hazard ratios were significantly greater than 1. Although it is possible that these observations may be caused by the continuous generation of relocalized duplicates from nonrelocalized duplicates over time rather than actual retention itself, a preliminary analysis of positive selection amongst the relocalized and nonrelocalized duplicate pairs from all species used in this study suggest that the data are more likely to be due to retention rather than a gradual accumulation of relocalized duplicates. Assuming that preferential retention of relocalized duplicates is due in part to some added benefit and thus subject to positive selection, we examined the data to see whether more relocalized duplicates exhibited evidence of positive selection over nonrelocalized duplicates. To obtain a reasonable sample size for the each of the Ks ranges used in our hazard ratio analysis, we combined all duplicates from all species used in this study. We then calculated the proportion of relocalized and nonrelocalized duplicates with a Ka/Ks > 1.5. Although Ka/Ks > 1 is typically the standard by which positive selection is measured, we chose 1.5 to give more weight to our initial analysis. For each of the Ks ranges used in this study, we found that significantly more relocalized duplicates have a Ka/Ks > 1.5 than nonrelocalized duplicates, a result consistent with preferential retention of relocalized duplicates rather than a gradual relocalization over time (supplementary table S3, Supplementary Material online).

**Table 3:** Hazard Ratios of Nonrelocalized vs. Relocalized Duplicate Genes in Some Eukaryotic Species for Ks Values Ranging from 0 < Ks < 1

Species	0 < Ks < 0.05	0 < Ks < 0.1	0 < Ks < 0.25	0 < Ks < 0.5	0 < Ks < 0.75	0 < Ks < 1
*Volvox carteri*	1.58*	1.30*	1.40**	1.32*	1.36**	1.13*
*Saccharomyces cervisiae*	1.73*	1.82**	3.80**	2.26**	2.23*	2.72**
*Caenorhabditis elegans*	0.92	0.69	1.36*	1.20*	1.23*	1.59**
*Drosophila rerio*	1.24**	1.52**	1.31**	1.35**	1.61**	1.55**
*Homo sapiens*	1.92**	1.56**	1.64**	1.38**	1.39**	1.27**
*Mus musculus*	1.26**	1.17**	1.09**	1.43**	1.46**	1.40**
*Phytophthora ramorum*	1.34**	1.38**	1.31**	1.21**	1.17**	1.13**
*Oryza sativa*	1.46**	1.24**	1.21**	1.22**	1.20**	1.24*

Note.—Hazard ratios = 1 indicate death rates between relocalized and nonrelocalized duplicates are equal. Hazard ratios > 1 indicate death rates of relocalized duplicates are lower than the death rates of nonrelocalized duplicates.

*Significant hazard ratios *P* < 0.05.

**Significant hazard ratios *P* < 0.001*.*

It is also possible that gene conversion could lead to biases in our death rate estimations with the appearance of fewer older (high Ks) nonrelocalized duplicates resulting in apparent lower retention rates over time. As gene conversion tends to occur in large gene families (>5 members), we removed all such duplicates by excluding those genes with more than five identifying matches (Lynch and Conery 2000) and then reanalyzed the data. Although specific patterns of retention for individual species changed as members of large gene families were removed, the overall results remained unchanged: Relocalized duplicate pairs had significantly higher retention than nonrelocalized duplicates. In fact, in this case, we found no instances in which nonrelocalized duplicates had significantly higher retention (supplementary table S4, Supplementary Material online).

The use of homology-based predictors like MultiLoc2 was another possible limitation with this analysis. Unfortunately, the performance of predictors that ignore homology would likely be inadequate for this type of study. To minimize potential biases introduced by homology, we reanalyzed the data by first removing all highly similar duplicate pairs (Ks < 0.01). This was done to eliminate the possibilty of erroneous predictions, which could lead to an overrepresentation of nonrelocalized duplicates with low Ks. When we compare the result of this analysis with the earlier results, we find the overall pattern intact: Relocalized duplicate pairs have significantly higher retention than nonrelocalized duplicates (supplementary tables S5 and S6, Supplementary Material online).

We observed that hazard ratios for individual species varied depending upon whether we used the entire or a subset of the data. One of the most dramatic differences we noticed was in *A. thaliana*. In the complete data set, we observed significant retention of relocalized duplicates but when corrected for gene conversion, we no longer saw such retention. In this particular case, it is possible that 1) gene conversion was biasing the data so that nonrelocalized duplicates appeared to have low Ks or that 2) a number of relocalized duplicates are located in large multigene families in *A. thaliana*. Removing them may have biased the results against retention of relocalized duplicates. Although we did not quantify our observations, we did note that a number of relocalized duplicates in *A. thaliana* did appear to belong to large multigene families involved in secondary metabolism. This is consistent with observations made by Heilmann et al. (2004).

**Fig. 1.—:**
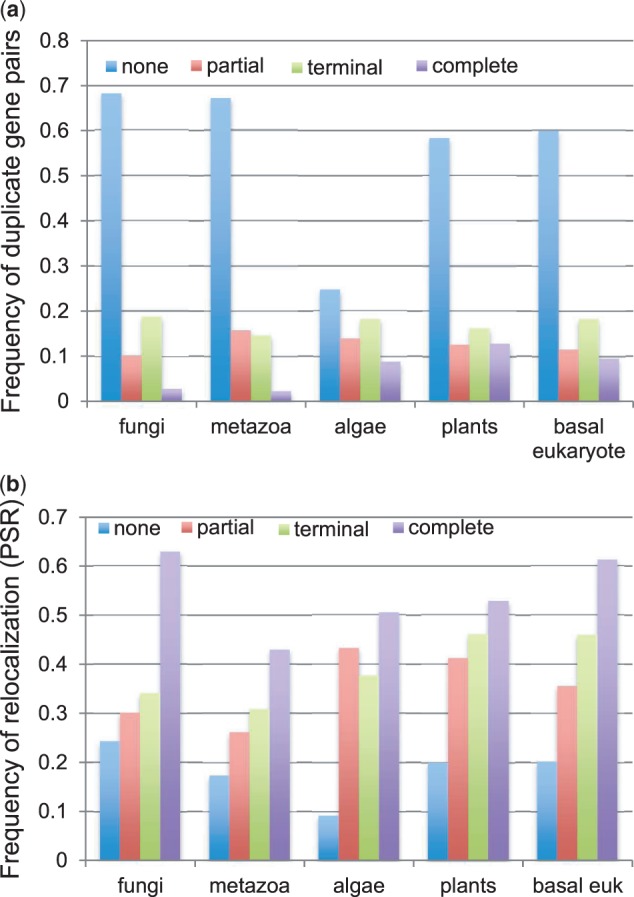
(*a*) Frequency of duplicate gene pairs with different NTP mutation types: None, Partial, Terminal, and Complete. In fungi, metazoans, plants, algae, and basal eukaryotes, the most common type of NTP mutation amongst duplicate gene pairs are base substitutions (None), whereas the least common were large deletions of 30 amino acids or more (Complete). Interestingly, plants have similar numbers of NTPs with terminal deletions as single base substitutions. (*b*) Frequency of PSR within each NTP mutation category. Proportion of relocalized duplicate pairs is highest for those pairs with complete NTP deletion/additions.

As part of our investigation, we also examined the mutation patterns in the NTP region of duplicate gene pairs. Although the most common types of NTP mutation in the analysis were duplicate pairs with base substitutions (None), it was the complete deletion/gain of the NTP that resulted in proportionately more subcellular relocalizations (fig. 1*a* and *b*). The higher frequency of relocalization associated with complete NTP indels compared with base substitutions is not necessarily surprising given the greater magnitude of the former type of mutation. Based on this observation, we speculate that different mechanisms of gene duplication may influence the manner in which the duplicate proteins relocalize. For example, whole-genome and large-scale segmental duplications would likely give rise to duplicates with intact NTPs. Products of these types of duplication events would probably relocalize through base substitutions (and/or indels), which we found to be very common in the NTP. On the other hand, small-scale duplications caused by mechanisms such as illegitimate crossing over have the potential to generate duplicates with complete NTP additions/deletions, which in turn are more likely to result in subcellular relocalizations. Although, in our study, we did not distingush between duplicates formed by whole-genome or segmental duplications, we did examine some species that have not had any documented whole-genome duplications (WGD) (e.g., *C. intestinalis*), and some that have had multiple WGD such as polyploidizations (e.g., *O. sativa*) (Blanc and Wolfe 2004). In both types of species, we found evidence to support the idea that relocalized duplicates tend to have higher retention than nonrelocalized duplicates. The purpose of this work was to examine general patterns of duplicate gene retention across eukaryotic genomes. A closer examination of these hazard ratios in specific species from the perspective of their unique genomic history as well as specific gene families is an area of future research.

How could PSR initially be advantageous enough to significantly increase retention? We can envision several scenarios. First, the ancestral function of duplicate genes may not change with relocalization but instead could allow that function to be carried out in different subcellular compartments. The existence of identical protein functions in different subcellular compartments is not a new concept and can be achieved through alternate transcription and translation, alternate splicing (Regev-Rudzki et al. 2005) and possibly through PSR of duplicate genes. Increased distribution of ancestral function in different compartments by PSR could be viewed as a type of subfuctionalization as described by Hughes (1994). Second, it is not unreasonable to think that a change in subcellular location can have an immediate and beneficial effect on function. Such a change in function has been demonstrated experimentally. For example, when the cytosolic protein IDE (insulin degrading enzyme) was engineered to relocalize to the mitochondria, it immediately changed its function from regulating plasma insulin levels to degrading peptides (Leissring et al. 2004). One possible example of PSR and a change in function is that of dioscorins. Dioscorins are essential storage proteins found in yam tubers (*Dioscorea* spp.). They are assumed to aggregrate in the vacuoles of storage cells due to the presence of a signal peptide at their N-terminus (Lu et al. 2012). Although they have signficant cDNA similarity to α-carbonic anhydrases (αCAHs), a gene family that participates in the reversible hydration of CO_2_ in mammals, dioscorins share many characteristics of plant storage proteins such as high amide content and solubility. Despite the lack of highly conserved histidines characteristic of αCAHs, it was found that dioscorins still possess the ability for αCAHs activity, leading researchers to conclude that dioscorins are a novel type of αCAHs (Lu et al. 2012). Given that αCAHs in C3 dicots are generally known to localize to the chloroplast and stroma (Moroney et al. 2001), it is possible that these storage proteins may be an example of neofunctionalization via PSR. Another possible example of neofunctionalization through PSR may be the nonmuscle myosin heavy chain isoforms MHC-A and MHC-B in *Xenopus.* These two isoforms are encoded by two duplicate genes and are known to have distinct subcellular localizations based on immunoflourescence microscopy. The isoforms were shown to have very different enzymatic activities leading researchers to suggest that these two MHC isoforms have distinct functions (Kelley et al. 1996). A literature search results in a plethora of examples which suggest that different subcellular locations of duplicate proteins are associated with diverse functions (Pidoux and Tasken 2010; Castellano and Santos 2011). In future, it would be interesting to document the functional categories of both relocalized and nonrelocalized duplicate pairs, along with their subcellular locations as it may reveal important evolutionary patterns about gene diversification via PSR. Although we cannot say with certainty that the example described earlier are cases of neofunctionalization by PSR without examining outgroups and information on ancestral function, in light of our data indicating significant retention of relocalized duplicate genes, such studies would be worth pursing.

Another advantage that can initially arise from relocalization may be to alleviate dosage effects. Relocalization of duplicate proteins may cause them to become functionally inactive due to changes in the metabolic environment of the cell. This could serve to reduce overexpression of these proteins and thereby restore normal protein dosage. Evolution of novel function through PSR and dosage effects is not necessarily mutually exclusive. In fact, they may work together in that initial retention through a reduction in dosage, may give a fraction of these duplicates the time needed to accumulate beneficial mutations resulting in advantageous functions.

## Conclusion

Several past studies have indicated the importance of PSR in the evolution of duplicate genes in vertebrates (Rosso et al. 2008; Kassahn et al. 2009). Here, we examined 56 species to see whether PSR plays a much wider role in eukaryotic genome evolution. The observation of significantly decreased death rates of relocalized duplicates in genomes spanning from single-celled eukaryotes to plants to mammals suggests that PSR is an important evolutionary process that may drive neofunctionalization in eukartyotes but yet has largely remained under-appreciated.

## Materials and Methods

Coding sequences (CDSs) of annotated genes from 59 species were downloaded from Ensembl (Flicek et al. 2011) release 62, and Ensembl Genomes release 9 (Kersey et al. 2010) (for a complete list of all species see supplementary table S1, Supplementary Material online). MySQL queries were used to obtain the complete set of CDSs for each species. Metazoan coding sequences (CDS) from Ensembl genes were obtained using the following SQL query from the public Ensembl MySQL server at ensembldb.ensembl.org, where ID was a variable interpolated by an ad hoc Perl script that repeated the query once for each species’ database.

SELECT m.stable_id,m.description,scds.sequence_cds FROM sequence_cds scds, member m WHERE m.member_id=scds.member_id AND m.genome_db_id=ID

CDSs for nonmetazoan species were obtained using the following SQL query from the online MySQL database at mysql.ebi.ac.uk in a similar manner.

SELECT stable_id, description, sequence_cds FROM member, sequence_cds WHERE sequence_cds.member_id = member.member_id AND member.genome_db_id = ID.

Gene descriptions and correspondences between gene, transcript and protein IDS were downloaded from Ensembl and Ensembl genome using XML-based queries using the Martservice utility of the BioMart (Haider et al. 2009) interface for each of the species. The two green algal CDS collections for *Chlamydomonas reinhartii* (Merchant et al. 2007) and *Volvox carteri* (Prochnik et al. 2010) were obtained from phytosome (Goodstein et al. 2012).

The functional units of these analyses are paralogous protein pairs that represent putative duplicated genes presumed to share a common ancestor in the species lineage. The CDS transcriptome for each species was processed to have one representative sequence for each coding gene. In cases where alternative transcripts were annotated, the longest CDS was selected to represent the gene. Each processed CDS transcriptome was conceptually translated to create a representation of the species’ proteome. Proteins pairs were initially identified through all versus all intraspecific Blast (Altschul et al. 1997) analysis. The initial criteria for selection on candidate pairs from Blast analysis were proteins that align with an expect (e value) of ≤1e−3 and a Blast score ratio of ≥0.33. The Blast score ratio (Vilella et al. 2009) takes into account the bit score for the protein pair A–B as relates to the self-score of each protein gets when BLASTed against itself, where
(1)
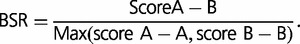



A larger BSR represents a higher quality of protein alignment in terms of length and sequence similarity. We used the threshold BSR of 0.33, as recommended by Vilella et al. (2009). Although all pairs were used in the clustering analysis (discussed later), we used proteins with ≥50% sequence identity along with at least 80% of their length for subcellular localization studies.

Protein pairs were then subjected to additional more stringent reciprocal filters to reduce spurious matches due to factors such as shared protein domains. Using criteria similar to those developed by Gu et al. (2002), we eliminated pairs whose alignment length was less than 80% of the total length of protein and, for peptides of length ≥150 amino acid residues, a minimum cutoff for percent sequence identity (*I*) of 30% was used. For peptides of length <150, the minimum *I* was calculated by using the method of Rost (1999):
(2)
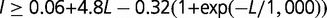

where *L* is the length of the alignment. This formula was derived from an empirical study that suggested that shorter peptides require a higher threshold for percent identity. Protein pairs that met all of the above criteria were retained for further analysis.

Proteins were clustered using a stringent double-linkage algorithm, in which filtered, reciprocal protein pairs for A, B, and C must all exist for proteins A, B, and C to be clustered. Resulting clusters are regarded as gene families. After this procedure, some proteins are represented in more than one cluster, which indicates that a nonreciprocal pair exists in the filtered set. Such pairs meet the e value and BSR thresholds but do not reciprocally pass the downstream filters, indicating a lower percentage identity or that they do not align more than 80% of protein length. Superclusters were formed by evaluating all protein pairs for single linkages and merging clusters where proteins were duplicated, until each protein was represented in only one cluster or supercluster. The family data, protein pair data and results of other analysis below were stored in a partially normalized MySQL database for future reference.

As gene conversion tends to occur in large gene families (>5 members), to minimize the potential effects from gene conversion, which could bias the death rates of nonrelocalized duplicate pairs, we ran all subsequent analyses on two data sets: 1) with all identified duplicate genes and 2) excluding all duplicates with more than five identifying matches (Lynch and Conery 2000).

The CDS sequences corresponding to protein pairs were assembled and each pair was analyzed for rates of synonymous (Ks) substitution. CDSs were translated and the proteins aligned with CLUSTALW (Thompson et al. 1994), which was then back-translated to the CDS alignment using an ad hoc BioPerl (Stajich et al. 2002) script. Ks was calculated using the yn00 program (Yang and Nielson 2000), which accounts for both the transition/transversion rate and codon usage biases.

Subcellular localization for individual proteins was predicted using Multiloc2 (Blum et al. 2009). Multiloc2 was used because it is capable of predicting localization in many subcellular compartments (specifically HighRes) and is trained for a greater variety of species groups. MultiLoc2 uses several subpredictors based on overall amino acid composition, identification of sorting signals, and detection of sequence motifs. Furthermore, the incorporation of phylogenetic profiles and GO (Gene Ontology) terms results in MultiLoc2 outperforming other comparable prediction systems in two benchmark studies done by Blum et al. (2009). One potential limitation in using MultiLoc2 is its use of homology. Such predictors could potentially cause erroneous subcellular predictions in pairs that have high similarity. Unfortunately, the performance of predictors, which ignore homology, would likely be inadequate for this type of study. To minimize these potential biases in MultiLoc2, we conducted the following survival analysis in two ways: 1) with all identified duplicate pairs and 2) with all duplicate pairs but those with a Ks < 0.01. By removing highly similar duplicate pairs, we remove those duplicates that are most likely to be problematic for MultiLoc2.

### N-Terminal Mutations

The NTP mutations were categorized as having 1) base pair substitutions (None); 2) indels at the terminal end (Terminal); 3) internal indels (Partial); and 4) complete deletions (Complete). For each of the five eukaryotic groups (fungi, metazoan, algae, plants, and basal eukaryotes), we determined the total number of duplicate pairs, the total number of duplicate pairs with each of the four types of mutations, and then calculated the frequency of each. We also calculated the frequency of relocalization of each mutation type by dividing the total number of relocalized duplicate pairs for each mutation category by the total number of relocalized pairs for each of the five eukaryotic groups.

### Statistical Analyses

The techniques of survival analysis include several parametric regression models (e.g., exponential, Weibull, log-logistic, and log-normal) and a semi-parametric model (Cox Proportional Hazard) to estimate the association between covariates and the distribution of the survival time or the response variable (Therneau and Grambsch 2000; Tableman and Kim 2004). The Cox Proportional Hazard model is currently the most widely used approach (Harrell 2001). In this study, we used the Cox proportional hazard model to compare the death rates associated with relocalized and nonrelocalized duplicate pairs (Therneau and Grambsch 2000). The model is defined as follows:
(3)
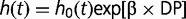

where *t* represents survival time (Ks) of relocalized and nonrelocalized duplicate pairs, *h*_0_(*t*) is called the baseline hazard, β is a coefficient, and DP represents duplicate pairs status (DP = 0 or relocalized duplicate pairs and DP = 1 or nonrelocalized duplicate pairs). The β coefficient is estimated by maximizing the partial likelihood function introduced by Cox (1972). The hazard ratio for DP = 1 and DP = 0 is defined as *h*_DP = 1_(*t*)/*h*_DP=0_(*t*) = e^1×β^/e^0×β ^= e^β^. The hazard ratio or e^β ^> 1 represents that the death rate of nonrelocalized duplicate pairs is higher as compared with relocalized duplicate pairs. The analyses were performed using an open source statistical software R (R Development Core Team 2011). All estimates and confidence intervals were obtained using the coxph function available in the survival package (Tableman and Kim 2004).

The Cox proportional hazard model does not assume that the gene duplication rate or the birth rate is constant. The only assumption is that the hazard in the comparison group (nonrelocalized duplicate genes) is a constant proportion of the hazard in the reference group (relocalized duplicate genes). Graphical checks of the overall adequacy of the Cox proportional hazard model was performed using the Cox–Snell residuals plot (Tableman and Kim 2004). The plots show that the model gave a reasonable fit to the data and therefore the proportionality assumption of the model is satisfied.

## Supplementary Material

Supplementary tables S1–S6 are available at *Genome Biology and Evolution* online (http://www.gbe.oxfordjournals.org/).

Supplementary Data
