# Supplementary material for: Protein Subcellular Relocalization Increases the Retention of Eukaryotic Duplicate Genes
Source: Genome Biol Evol. 2013 Nov 20;5(12):2402–9. doi: 10.1093/gbe/evt183 (PMC3879971; doi:10.1093/gbe/evt183)
Supplement: Supplementary Data [file supp_5_12_2402__index.html]

Protein subcellular relocalization increases the retention of eukaryotic duplicate genes — Protein Subcellular Relocalization Increases the Retention of Eukaryotic Duplicate Genes — Supplementary Data 

# Protein Subcellular Relocalization Increases the Retention of Eukaryotic Duplicate Genes

## Supplementary Data

files

**Files in this Data Supplement:**

- Supplementary Data - docx file
- Supplementary Data - docx file
- Supplementary Data - docx file
- Supplementary Data - docx file
- Supplementary Data - docx file
- Supplementary Data - docx file
