# Supplementary material for: Protein Subcellular Relocalization Increases the Retention of Eukaryotic Duplicate Genes
Source: Genome Biol Evol. 2013 Nov 20;5(12):2402–9. doi: 10.1093/gbe/evt183 (PMC3879971; doi:10.1093/gbe/evt183)
Supplement: Supplementary Data [file supp_evt183_Supplementary_Table_S1_List_of_Species.docx]

**Table S1.** Percentage of duplication and PSR

| **Group** | **Species** | **%DG** | **%RDG** |
| --- | --- | --- | --- |
| Algae | *Chlamydomonas reinhardtii* | 9.43 | 20.7 |
| Algae | *Volvox carteri* | 10.4 | 32.5 |
| Fungi | *Aspergillus nidulans* | 11 | 30.8 |
| Fungi | *Fusarium oxysporum* | 23.4 | 12.8 |
| Fungi | *Saccharomyces cerevisiae* | 16.1 | 28.6 |
| Fungi | *Schizosaccharomyces pombe* | 11.7 | 16.6 |
| Metazoa | *Bos taurus* | 37.4 | 26.1 |
| Metazoa | *Caenorhabditis elegans* | 27.6 | 22 |
| Metazoa | *Callithrix jacchus* | 34.4 | 19.3 |
| Metazoa | *Canis familiaris* | 31.6 | 16.7 |
| Metazoa | *Ciona intestinalis* | 18.2 | 11.4 |
| Metazoa | *Ciona savignyi* | 13.4 | 15.2 |
| Metazoa | *Danio rerio* | 46.8 | 28.5 |
| Metazoa | *Dasypus novemcinctus* | 12.2 | 11.2 |
| Metazoa | *Dipodomys ordii* | 13.9 | 11.7 |
| Metazoa | *Drosophila melanogaster* | 17.8 | 10.3 |
| Metazoa | *Echinops telfairi* | 15 | 17.4 |
| Metazoa | *Equus caballus* | 38.7 | 9 |
| Metazoa | *Erinaeus europaeus* | 11.5 | 17.5 |
| Metazoa | *Felis catus* | 9.6 | 18 |
| Metazoa | *Gallus gallus* | 81.6 | 22.6 |
| Metazoa | *Gorilla gorilla* | 10.2 | 22.6 |
| Metazoa | *Homo sapiens* | 32.6 | 30.4 |
| Metazoa | *Macaca mulatta* | 34.1 | 23.7 |
| Metazoa | *Macropus eugenii* | 14.4 | 10.7 |
| Metazoa | *Microcebus murinus* | 13.7 | 20.9 |
| Metazoa | *Monodelphis domestica* | 37.7 | 13.8 |
| Metazoa | *Mus musculus* | 41.4 | 17 |
| Metazoa | *Nomascus leucogenys* | 25.4 | 23.3 |
| Metazoa | *Ochotona princeps* | 14 | 20.6 |
| Metazoa | *Ornithorhynchus anatinus* | 20.7 | 57.3 |
| Metazoa | *Oryzias latipes* | 38.8 | 21.8 |
| Metazoa | *Pan troglodytes* | 28.5 | 28 |
| Metazoa | *Pongo abelii* | 28.5 | 22.6 |
| Metazoa | *Procavia capensis* | 11.9 | 14.6 |
| Metazoa | *Pteropus vampyrus* | 19.2 | 18.1 |
| Metazoa | *Rattus norvegicus* | 39.3 | 17.7 |
| Metazoa | *Sorex araneus* | 13.5 | 27.5 |
| Metazoa | *Spermophilus tridecemlineatus* | 10.2 | 29.4 |
| Metazoa | *Taeniopygia guttata* | 27.4 | 14 |
| Metazoa | *Takifugu rubripes* | 41.2 | 17.9 |
| Metazoa | *Tarsius syrichta* | 12.6 | 24.6 |
| Metazoa | *Tetradon nigroviridis* | 36.8 | 18.7 |
| Metazoa | *Tupaia belangeri* | 13.6 | 15.6 |
| Metazoa | *Tursiops truncatus* | 18.7 | 18.8 |
| Metazoa | *Vicugna pacos* | 11.5 | 14.6 |
| Metazoa | *Xenopus tropicalis* | 40.2 | 24.7 |
| Plant | *Arabidopsis thaliana* | 50.7 | 27.8 |
| Plant | *Brachypodium distachyon* | 40.2 | 34.5 |
| Plant | *Oryza sativa* | 38.1 | 35.3 |
| Plant | *Physcomitrella patens* | 62.1 | 28.5 |
| Plant | *Populus trichocarpa* | 58.8 | 30.1 |
| Plant | *Sorghum bicolor* | 40.6 | 34.1 |
| Plant | *Vitis vinifera* | 31.5 | 31.8 |
| Plant | *Zea mays* | 33 | 26.2 |
| Basal Euk | *Dictyostelium discoideum* | 62.9 | 7 |
| Basal Euk | *Phaeodactylum tricornutum* | 13.4 | 31 |
| Basal Euk | *Phytophthora ramorum* | 30.2 | 33 |
| Basal Euk | *Thalassiosira pseudonana* | 11.4 | 23.3 |

%DG - % of duplicated genes

%RDG - % of relocalized duplicated genes as predicted by MultiLoc 2.
