# Supplementary material for: Protein Subcellular Relocalization Increases the Retention of Eukaryotic Duplicate Genes
Source: Genome Biol Evol. 2013 Nov 20;5(12):2402–9. doi: 10.1093/gbe/evt183 (PMC3879971; doi:10.1093/gbe/evt183)
Supplement: Supplementary Data [file supp_evt183_Supplementary_Table_S2_Hazard_Ratios.docx]

**Table S2 Hazard ratios for Ks values ranging between 0 and 1.**

Values shaded green represent hazard ratios significantly >1. Values shaded purple represent hazard ratios significantly <1. Unshaded values are not significant. The higher proportion of significant hazard ratios>1 (green) suggests the death rates of relocalized duplicate pairs is significantly lower than death rates of non-relocalized duplicates in most of the eukaryotic genomes examined in this study.

| **Species** | **0<Ks<0.05** | **0<Ks<0.1** | **0<Ks<0.25** | **0<Ks<0.5** | **0<Ks<0.75** | **0<Ks<1** |
| --- | --- | --- | --- | --- | --- | --- |
| *C. reinhardtii* | 1.34 * | 1.01 | 0.81* | 0.72 ** | 0.84 ** | 0.90 |
| *V. carteri* | 1.58 * | 1.30 * | 1.40 ** | 1.32 * | 1.36** | 1.13 |
| *A. nidulans* | 2.37 * | 1.32 | 1.70 * | 2.79 ** | 2.79 ** | 1.81 * |
| *F. oxysporum* | 1.04 | 1.80 ** | 1.38 ** | 1.45 ** | 1.39 ** | 1.35 ** |
| *S. cerevisiae* | 1.73 * | 1.82 **` | 3.80 ** | 2.26 ** | 2.23 ** | 2.72 ** |
| *S. pombe* | NA | NA | 8.93 ** | 2.03 * | 1.56 | 1.15 |
| *B. taurus* | 1.67 * | 1.82 ** | 1.80 ** | 1.23 * | 1.10 * | 0.96 |
| *C. elegans* | 0.92 | 0.69 | 1.36 * | 1.20 * | 1.23 * | 1.59 ** |
| *C. jacchus* | 1.64 * | 1.32 * | 1.52 ** | 1.55 ** | 1.56 ** | 1.50 ** |
| *C. familiaris* | 1.23 | 1.17 | 1.52 ** | 1.46 ** | 1.24 * | 0.95 |
| *C. intestinalis* | 1.28 | 1.50 * | 1.35 | 1.50 * | 1.39 * | 1.18 |
| *C. savignyi* | 1.72 | 0.52 | 1.21 | 1.08 | 1.04 | 1.26 |
| *D. rerio* | 1.24 ** | 1.52 ** | 1.31 ** | 1.35 ** | 1.61 ** | 1.55 ** |
| *D.novemcinctus* | 0.35 * | 0.76 | 0.62 * | 0.84 | 0.87 | 0.76 |
| *D.ordii* | NA | 2.25 * | 0.98 | 1.01 | 0.74 * | 0.67 * |
| *D.melanogaster* | NA | 3.21 * | 2.27 * | 2.33 * | 1.81 * | 1.77 * |
| *E. telfairi* | 1.21 | 1.43 | 0.88 | 1.06 | 1.02 | 0.88 |
| *E. caballus* | 0.46 * | 1.72 ** | 1.42 ** | 1.37 ** | 1.47 ** | 1.27 ** |
| *E. europaeus* | 1.22 | 0.67 | 1.62 | 0.96 | 0.94 | 1.00 |
| *F.catus* | NA | NA | 3.02 | 1.57 | 1.39 | 1.34 |
| *G.gallus* | 2.73 * | 1.33 | 1.66 ** | 1.02 | 0.71 ** | 0.60 ** |
| *G. gorilla* | 1.307 * | 1.06 | 1.307 ** | 1.309 ** | 1.168 * | 1.167 * |
| *H. sapiens* | 1.92 ** | 1.56 ** | 1.64 ** | 1.38 ** | 1.39 ** | 1.27 ** |
| *M.mulatta* | 1.43 * | 1.73 ** | 1.61 ** | 1.39 ** | 1.37 ** | 1.29 ** |
| *M. eugenii* | 1.22 | 1.44 | 1.60 * | 0.75 | 1.09 | 1.02 |
| *M. murinus* | 1.12 | 0.83 | 2.26 ** | 1.72 ** | 1.32 | 0.99 |
| *M.domestica* | 1.52 * | 1.25 | 0.84 | 1.16 | 1.42 ** | 1.12 |
| *M.musculus* | 1.26 ** | 1.17 ** | 1.09 ** | 1.43 ** | 1.46 ** | 1.394 ** |
| *N. leucogenys* | 0.90 | 1.78 * | 1.24 | 1.51 * | 1.40 * | 1.19 |
| *O.princeps* | NA | 1.70 * | 2.38 * | 1.91 * | 1.35 * | 1.03 |
| *O. anatinus* | 1.21 | 1.11 | 1.11 | 1.17 * | 1.14 | 1.10 |
| *O. latipes* | 1.20 | 1.12 | 1.24 * | 1.32 ** | 1.56 ** | 1.26 ** |
| *P. troglodytes* | 0.90 | 1.78 * | 1.24 | 1.51 * | 1.40 * | 1.19 |
| *P.abelii* | 1.05 | 1.07 | 1.26 * | 1.44 ** | 1.33 ** | 1.19 * |
| *P.capensis* | NA | 3.34 * | 1.03 | 1.10 | 1.30 | 1.12 |
| *P. vampyrus* | 1.12 | 1.01 | 1.71 * | 2.00 ** | 1.38 * | 1.30 * |
| *R.norvegicus* | 1.24 | 1.37 * | 1.41 ** | 1.16 ** | 1.07 * | 0.98 |
| *S. araneus* | 1.03 | 1.12 | 0.99 | 0.71 ** | 0.63 ** | 0.73 ** |
| *S. tridecemlineatus* | 1.45 | 1.48 | 1.55 ** | 1.08 | 0.97 | 0.73 * |
| *T. guttata* | 1.00 | 1.73 ** | 1.31 ** | 0.96 | 0.76 ** | 0.79 ** |
| *T. rubripes* | 0.82 | 1.60 | 1.82 * | 1.11 | 1.35 | 1.35 |
| *T. syrichta* | 2.02 * | 0.86 | 1.44 * | 1.55 * | 1.31 | 1.12 |
| *T. nigroviridis* | 1.41 | 0.882 | 1.03 | 1.16 | 1.12 | 1.12 |
| *T. belangeri* | 1.20 | 1.37 ** | 1.18 ** | 1.15 * | 1.03 | 1.03 |
| *T. truncatus* | 0.67 | 1.13 | 1.83 | 0.67 | 1.13 | 1.83 |
| *V. pacos* | 0.19 * | 2.08 | 1.29 | 1.73 | 1.30 | 0.89 |
| *X. tropicalis* | 0.95 | 1.13 | 0.94 | 0.85* | 0.79 ** | 0.76 ** |
| *A. thaliana* | 1.05 | 1.55 ** | 1.72 ** | 1.72 ** | 1.41 ** | 1.31 ** |
| *B. distachyon* | 1.4 | 0.98 | 1.20 * | 1.15 * | 1.15 * | 1.15 * |
| *O. sativa* | 1.46 ** | 1.24 ** | 1.21 ** | 1.22 ** | 1.20 ** | 1.24 ** |
| *P. patens* | 0.76 ** | 0.81 ** | 1.21 ** | 9.0 ** | 1.02 * | 1.04 ** |
| *P. trichocarpa* | 1.15 * | 1.14 * | 1.01 | 1.15 ** | 1.26 ** | 1.33 ** |
| *S. bicolor* | 1.15 * | 1.26 ** | 1.12 * | 1.18 ** | 1.14 ** | 1.13 ** |
| *V. vinifera* | 1.09 | 1.23 * | 1.25 ** | 1.40 ** | 1.33 ** | 1.25 ** |
| *Z. mays* | 1.11 * | 1.36 ** | 1.23 ** | 1.18 ** | 1.21 ** | 1.29 ** |
| *D. discoideum* | 1.59 * | 1.36 * | 1.48 ** | 1.50 ** | 1.31 ** | 1.13 |
| *P. tricornutum* | 1.18 | 1.40 | 1.63 * | 1.75 ** | 1.45 * | 1.52 ** |
| *P. ramorum* | 1.34 * | 1.38 ** | 1.31 ** | 1.21 ** | 1.17 ** | 1.13 * |
| *T. pseudonana* | 1.04 | 1.05 | 0.74 | 0.79 | 0.95 | 0.98 |

* P<0.05

**P<0.001

NA: No data is available
