# Supplementary material for: Protein Subcellular Relocalization Increases the Retention of Eukaryotic Duplicate Genes
Source: Genome Biol Evol. 2013 Nov 20;5(12):2402–9. doi: 10.1093/gbe/evt183 (PMC3879971; doi:10.1093/gbe/evt183)
Supplement: Supplementary Data [file supp_evt183_Supplementary_Table_S3_Positive_Selection_Byun_Singh.docx]

**Supplementary Table S3. Proportion of relocalized duplicate (RD) and non-relocalized duplicate (ND) pairs with Ka/Ks≥1.5.** To look for evidence of positive selection, nucleotide sequences corresponding to protein pairs were assembled and each pair was analyzed for rates of synonymous (Ks) and non-synonymous (Ka) substitution. CDSs were translated and the proteins aligned with CLUSTALW (Thompson et al.,1994), which was then back-translated to the CDS alignment using an *ad hoc* BioPerl (Stajich et al, 2002) script. Ka, Ks, and the Ka/Ks ratio were calculated using a modified version (J. Stajich, personal communication) of the yn00 program (Yang and Nielson 2000), which accounts for both the transition/tranversion rate and codon usage biases. We combined all duplicates from all species used in this study in order to obtain a reasonable sample size for the Ks ranges shown below. Using a two proportion Z-test, in all instances, we found that significantly more relocalized duplicate pairs had a Ka/Ks≥1.5, an observation consistent with preferential retention of relocalized duplicates rather than a gradual accumulation of relocalized duplicates.

|  |
| --- |

**Species 0<Ks< 0.01 0<Ks< 0.05 0<Ks< 0.1 0<Ks< 0.25 0<Ks< 0.5 0<Ks< 0.75 0<Ks< 1.0**

|  |
| --- |

RD 0.37 0.27 0.17 0.075 0.052 0.047 0.045

ND 0.12 0.14 0.094 0.056 0.043 0.040 0.038

|  |
| --- |

Z 41.8** 49.7** 44.7** 22.4** 14.7** 13.1** 12.3**

** indicates significant z test p<0.001
