# Supplementary material for: Protein Subcellular Relocalization Increases the Retention of Eukaryotic Duplicate Genes
Source: Genome Biol Evol. 2013 Nov 20;5(12):2402–9. doi: 10.1093/gbe/evt183 (PMC3879971; doi:10.1093/gbe/evt183)
Supplement: Supplementary Data [file supp_evt183_Supplementary_Table_S4_Hazard_Ratios_Byun_Singh.docx]

**Table S4 Hazard ratios for Ks values ranging between 0.01 and 1. Analysis includes all duplicate genes except highly similar pairs Ks<0.01.**

Values shaded green represent hazard ratios significantly >1. Values shaded purple represent hazard ratios significantly <1. Unshaded values are not significant. The higher proportion of significant hazard ratios>1 (green) suggests the death rates of relocalized duplicate pairs is significantly lower than death rates of non-relocalized duplicates in most of the eukaryotic genomes examined in this study.

| **Species** | **0.01<Ks<0.1** | **0.01<Ks<0.25** | **0.01<Ks<0.5** | **0.01<Ks<0.75** | **0.01<Ks<1** |
| --- | --- | --- | --- | --- | --- |
| *C. reinhardtii* | 0.98 | 0.794* | 0.711* | 0.833* | 0.9 |
| *V. carteri* | 1.243 | 1.347* | 1.274* | 1.316* | 1.088 |
| *A. nidulans* | 0.182* | 0.907 | 2.055* | 2.055* | 1.068 |
| *F. oxysporum* | 1.691** | 1.256* | 1.336** | 1.276** | 1.248* |
| *S. cerevisiae* | 1.995* | 3.774* | 2.12* | 2.09* | 2.579* |
| *S. pombe* | NA | 5.748* | 0.937 | 0.641 | 0.418* |
| *B. taurus* | 1.661** | 1.67** | 1.147** | 1.015 | 0.886* |
| *C. elegans* | 0.602* | 1.306* | 1.186* | 1.224* | 1.58** |
| *C. jacchus* | 1.238* | 1.471** | 1.505** | 1.518** | 1.453** |
| *C. familiaris* | 1.037 | 1.442** | 1.392** | 1.179* | 0.9 |
| *C. intestinalis* | 1.569* | 1.374 | 1.505* | 1.388* | 1.159 |
| *C. savignyi* | 0.472* | 1.174 | 1.055 | 1.017 | 1.232 |
| *D. rerio* | 1.509** | 1.296** | 1.341** | 1.592* | 1.533* |
| *D.novemcinctus* | 0.846 | 0.656* | 0.882 | 0.901 | 0.79 |
| *D.ordii* | 2.045* | 0.908 | 0.97 | 0.711* | 0.645** |
| *D.melanogaster* | 2.184 | 1.542 | 1.692* | 1.239 | 1.253 |
| *E. telfairi* | 1.367 | 0.857 | 1.043 | 1.005 | 0.865 |
| *E. caballus* | 1.709** | 1.411** | 1.366** | 1.468** | 1.269** |
| *E. europaeus* | 0.607 | 1.575* | 0.975 | 0.922 | 0.99 |
| *F.catus* | NA | 2.945 | 1.518 | 1.35 | 1.312 |
| *G.gallus* | 1.312 | 1.659** | 1.015 | 0.709** | 0.601 |
| *G. gorilla* | 0.965 | 1.261* | 1.273** | 1.135 | 1.137* |
| *H. sapiens* | 1.447** | 1.501** | 1.27** | 1.289** | 1.168** |
| *M.mulatta* | 1.716** | 1.587** | 1.366** | 1.351** | 1.272** |
| *M. eugenii* | 1.401 | 1.581* | 0.742 | 1.083 | 1.015 |
| *M. murinus* | 0.857 | 2.304** | 1.71* | 1.304 | 0.966 |
| *M.domestica* | 1.14 | 0.787* | 1.109 | 1.374** | 1.08 |
| *M.musculus* | 1.125** | 1.046* | 1.396** | 1.43** | 1.361** |
| *N. leucogenys* | 1.669 | 1.193 | 1.473* | 1.372* | 1.164 |
| *O.princeps* | 1.586 | 2.312* | 1.863* | 1.32 | 1.006 |
| *O. anatinus* | 1.854* | 1.193 | 1.264** | 1.272** | 1.193** |
| *O. latipes* | 1.097 | 1.101 | 1.159* | 1.128 | 1.085 |
| *P. troglodytes* | 1.038 | 1.196 | 1.297** | 1.532** | 1.24** |
| *P.abelii* | 1.016 | 1.225* | 1.415** | 1.307** | 1.165* |
| *P.capensis* | 3.267* | 1.018 | 1.094 | 1.289 | 1.109 |
| *P. vampyrus* | 1.027 | 1.734* | 2.011** | 1.385* | 1.297 |
| *R.norvegicus* | 1.365** | 1.399** | 1.154** | 1.065* | 0.976 |
| *S. araneus* | 1.119 | 0.982 | 0.703** | 0.626** | 0.73** |
| *S. tridecemlineatus* | 1.443 | 1.532* | 1.071 | 0.959 | 0.725* |
| *T. guttata* | 1.702** | 1.297* | 0.949 | 0.754** | 0.78** |
| *T. rubripes* | 1.795 | 1.851* | 1.098 | 1.343 | 1.341 |
| *T. syrichta* | 0.665 | 1.314 | 1.442* | 1.215 | 1.032 |
| *T. nigroviridis* | 0.737 | 0.931 | 1.07 | 1.036 | 1.046 |
| *T. belangeri* | 1.3** | 1.147* | 1.122* | 1.004 | 1.004 |
| *T. truncates* | 0.996 | 1.713 | 1.908* | 0.786 | 1.064 |
| *V. pacos* | 2.973 | 1.365 | 1.795 | 1.327 | 0.904 |
| *X. tropicalis* | 1.134 | 0.943 | 0.848* | 0.793** | 0.765** |
| *A. thaliana* | 1.434* | 1.461** | 1.205** | 1.123** | 1.064* |
| *B. distachyon* | 0.981 | 1.192* | 1.146* | 1.148* | 1.143* |
| *O. sativa* | 1.138** | 1.152** | 1.175** | 1.15** | 1.195** |
| *P. patens* | 0.827** | 1.231** | 0.908** | 1.023** | 1.042** |
| *P. trichocarpa* | 1.117* | 0.999 | 1.145** | 1.253** | 1.327** |
| *S. bicolor* | 1.16* | 1.073 | 1.145** | 1.111* | 1.108** |
| *V. vinifera* | 1.208* | 1.233** | 1.385** | 1.32** | 1.232** |
| *Z. mays* | 1.33** | 1.203** | 1.154** | 1.189** | 1.265** |
| *D. discoideum* | 1.082 | 1.263* | 1.326* | 1.355** | 1.155 |
| *P. tricornutum* | 1.343 | 1.572* | 1.672* | 1.316* | 1.402* |
| *P. ramorum* | 1.312** | 1.255** | 1.155* | 1.124* | 1.126* |
| *T. pseudonana* | 1.037 | 0.706 | 0.778 | 0.949 | 0.983 |

* P<0.05

**P<0.001

NA: No data is available
