# Supplementary material for: Protein Subcellular Relocalization Increases the Retention of Eukaryotic Duplicate Genes
Source: Genome Biol Evol. 2013 Nov 20;5(12):2402–9. doi: 10.1093/gbe/evt183 (PMC3879971; doi:10.1093/gbe/evt183)
Supplement: Supplementary Data [file supp_evt183_Supplementary_Table_S5_Hazard_Ratios_Byun_Singh.docx]

**Table S6 Hazard ratios for Ks values ranging between 0.01 and 1. All duplicates from large gene families (>5 members removed) and highly similar pairs (Ks<0.01) removed**

Values shaded green represent hazard ratios significantly >1. Unshaded values are not significant. The higher proportion of significant hazard ratios>1 (green) suggests the death rates of relocalized duplicate pairs is significantly lower than death rates of non-relocalized duplicates in most of the eukaryotic genomes examined in this study.

| **Species** | **0.01<Ks<0.1** | **0.01<Ks<0.25** | **0.01<Ks<0.5** | **0.01<Ks<0.75** | **0.01<Ks<1** |
| --- | --- | --- | --- | --- | --- |
| *C. reinhardtii* | 0.784 | 1.379 | 1.535* | 1.471* | 1.52* |
| *V. carteri* | 1.306 | 1.407* | 1.193 | 1.028 | 1.073 |
| *A. nidulans* | NA | 2.356 | 3.474* | 3.474* | 1.063 |
| *F. oxysporum* | 1.854* | 1.229 | 1.387* | 1.409* | 1.744** |
| *S. cerevisiae* | NA | 1.336 | 0.738 | 1.035 | 1.083 |
| *S. pombe* | NA | 7.224* | 1.093 | 0.697 | 0.429 |
| *B. taurus* | 1.307 | 1.346 | 1.369* | 1.454* | 1.589** |
| *C. elegans* | 1.069 | 1.354 | 1.066 | 1.046 | 1.193 |
| *C. jacchus* | 1.048 | 1.286 | 1.556** | 1.806** | 1.554** |
| *C. familiaris* | 0.95 | 0.955 | 0.851 | 1.466* | 1.087 |
| *C. intestinalis* | 1.372 | 1.588* | 1.666* | 1.446* | 1.344 |
| *C. savignyi* | 0.533 | 0.963 | 0.967 | 1.034 | 1.573* |
| *D. rerio* | 1.475* | 1.281 | 1.116 | 1.263 | 1.152 |
| *D.novemcinctus* | 1.889 | 2.685* | 2.373* | 2.066* | 1.836* |
| *D.ordii* | NA | 2.67 | 0.984 | 1.275 | 1.004 |
| *D.melanogaster* | 2.506 | 1.013 | 1.062 | 1.042 | 1.384 |
| *E. telfairi* | 1.121 | 1.079 | 1.53 | 1.646* | 1.378 |
| *E. caballus* | 1.251 | 1.395 | 1.292 | 1.919* | 1.517* |
| *E. europaeus* | 0.591 | 2.802* | 1.479 | 1.96* | 1.467 |
| *F.catus* | NA | 1.714 | 1.251 | 1.445 | 1.314 |
| *G.gallus* | 1.825 | 2.464 | 1.964 | 3.307* | 1.357 |
| *G. gorilla* | 1.102 | 1.263 | 1.262 | 1.363* | 1.4* |
| *H. sapiens* | 1.093 | 1.051 | 1.185 | 1.355* | 1.282* |
| *M.mulatta* | 1.072 | 1.538* | 1.483* | 1.456* | 1.368* |
| *M. eugenii* | NA | 2.974* | 1.262 | 0.948 | 1.175 |
| *M. murinus* | 1.355 | 2.405* | 1.365 | 1.201 | 1.219 |
| *M.domestica* | 1.339 | 1.01 | 1.244 | 1.263 | 1.247 |
| *M.musculus* | 1.301 | 1.746** | 1.676** | 1.501** | 1.38* |
| *N. leucogenys* | 2.02* | 1.128 | 1.139 | 1.44* | 1.378 |
| *O.princeps* | NA | 7.739 | 2.376* | 1.905 | 1.619 |
| *O. anatinus* | NA | NA | 1.697 | 1.513 | 1.267 |
| *O. latipes* | 1.23 | 1.218 | 1.296 | 1.727* | 1.212 |
| *P. troglodytes* | 1.112 | 1.022 | 1.306 | 1.581* | 1.381* |
| *P.abelii* | 1.437* | 1.351* | 1.47** | 1.376** | 1.107 |
| *P.capensis* | 9.149* | 1.154 | 1.576 | 1.049 | 0.863 |
| *P. vampyrus* | 1.171 | 1.934* | 1.113 | 1.068 | 0.934 |
| *R.norvegicus* | 1.134 | 1.348* | 1.529* | 1.568** | 1.716** |
| *S. araneus* | 0.816 | 1.198 | 1.56 | 2.71* | 2.336* |
| *S. tridecemlineatus* | 1.748 | 1.947* | 1.275 | 1.096 | 0.993 |
| *T. guttata* | 1.397 | 1.571 | 1.413 | 1.294 | 1.321 |
| *T. rubripes* | 0.738 | 3.412* | 1.304 | 0.983 | 1.59 |
| *T. syrichta* | NA | 1.776 | 2.824* | 1.263 | 1.177 |
| *T. nigroviridis* | 0.649 | 0.889 | 1.405 | 1.114 | 1.351 |
| *T. belangeri* | NA | 2.244* | 2.5** | 2.252* | 1.697* |
| *T. truncatus* | 1.517 | 1.134 | 1.845 | 1.38 | 2.228* |
| *V. pacos* | NA | 1.797 | 1.838 | 2.251 | 1.003 |
| *X. tropicalis* | 1.948 | 0.842 | 1.74* | 1.542 | 1.248 |
| *A. thaliana* | 0.925 | 1.172 | 1.157 | 1.081 | 0.96 |
| *B. distachyon* | 1.181 | 1.231* | 1.227** | 1.167* | 1.133* |
| *O. sativa* | 1.083 | 1.275 | 0.995 | 1.078 | 1.069 |
| *P. patens* | 1.052 | 1.043 | 1.081* | 1.062 | 1.077* |
| *P. trichocarpa* | 1.084 | 1.104 | 1.201* | 1.207* | 1.221** |
| *S. bicolor* | 1.468* | 1.208* | 1.334** | 1.256* | 1.176* |
| *V. vinifera* | 1.214* | 1.103* | 1.1* | 1.228** | 1.273** |
| *Z. mays* | 0.835 | 0.922 | 1.335 | 1.545* | 1.191 |
| *D. discoideum* | 1.181 | 1.231* | 1.227** | 1.167* | 1.133* |
| *P. tricornutum* | 1.291 | 1.581* | 1.652* | 1.32 | 1.928* |
| *P. ramorum* | 1.18 | 1.415* | 1.18 | 1.234 | 1.233 |
| *T. pseudonana* | 1.109 | 0.95 | 1.47 | 1.494 | 1.319 |

* P<0.05

**P<0.001

NA: No data is available
